# Effect of spaceflight on the phenotype and proteome of *Escherichia coli*

**DOI:** 10.1515/biol-2022-0576

**Published:** 2023-02-28

**Authors:** Yu Liu, Chong Xu, Guangxian Zhao, Yanji Wang, Yuanbing Zhu, Yishu Yin, Jiaping Wang, Yongzhi Li

**Affiliations:** China Astronaut Research and Training Center, Beijing 100094, China; Xingcheng Special Duty Sanatorium, Xingcheng 125105, China

**Keywords:** spaceflight, proteomics, *Escherichia coli*

## Abstract

Microbial safety has become a research hotspot with the development of manned space technology. *Escherichia coli* is a conditional pathogen that can cause infectious diseases. Therefore, it is necessary to study the influence of the space environment on *E. coli*. Phenotypic experiments including growth curves, morphology, and environmental resistance experiment were used to study the phenotypic changes of *E. coli* after exposure to the space environment for 12 days carried by the “SJ-10” satellite. Tandem mass tag was used to assess the proteome change of *E. coli.* We found that the survival rate of *E. coli* in the spaceflight group was decreased when cultivated in acidic and high-salt environments. Proteomic analysis identified 72 downregulated proteins involved in chemotaxis, intracellular pH elevation, glycolate catabolic process, and glutamate metabolic process in the spaceflight group. Meanwhile, only one protein mtr that was involved in the uptake of tryptophan in *E. coli* was upregulated in the spaceflight group. Our research showed that proteomics results can explain phenotypic results, which demonstrated the successful application of proteomics in mechanism research. Our data provide a comprehensive resource for understanding the effect of the space environment on *E. coli*.

## 1 Introduction

*Escherichia coli* is a common Gram-negative bacterium that mainly exists in the intestines of humans and animals. It is an opportunistic pathogen, which can cause gastrointestinal infections in humans under certain conditions. In recent years, with the development of manned space technology, astronauts have been working longer and longer in space. Usually, bacteria will accompany humans into space [1]. Due to the existence of weightlessness, radiation, vacuum, and extreme temperature environments in space, the phenotype of bacteria including the growth rate, antibiotic resistance, anti-oxidation, biofilm formation, and virulence can be changed after being exposed to the space environment [1,2,3]. Conversely, the astronaut’s immune system is dysregulated [4,5]. Therefore, the existence of conditional pathogens may threaten the health of astronauts in the space environment. It is important to understand the phenotype and molecular changes of bacteria to ensure the health of astronauts during long-term space activities.

Proteins are the executor of most biological functions. With the development of proteomic technology, high-throughput protein identification became available for understanding global gene regulation. Tandem mass tag (TMT) technology is one of the most commonly used isobaric labeling technologies in mass spectrometry (MS) for the quantification of proteins present in different samples. The advantages of this method are high quantitative accuracy and high throughput, which enable the measurement of up to 16 samples simultaneously in a single run [6]. In this study, *E. coli* was carried by the SJ-10 returnable scientific experiment satellite in orbit for 12 days. The matched controls were cultured on the ground. The phenotypic changes of *E. coli* were explored, and the 6-plex TMT was used to compare the proteome of *E. coli* after exposure to the space environment. We found that proteomics results can explain phenotypic changes in the spaceflight group. This study should provide an important reference for understanding the effects of spaceflight on *E. coli*, which will contribute to keeping astronauts healthy during space flight.

## 2 Materials and methods

### 2.1 Strains and growth conditions

*E. coli* K12 strain (CGMCC 1.2389) was purchased from China General Microbiological Culture Collection Center (CGMCC). After cultivating *E. coli* to the exponential phase (OD_600_ = 1), 20 µL of bacteria was inoculated into an lysogeny broth (LB) agar slant culture medium. After being incubated at 37°C for 16 h, samples were cultured in space for 12 days, carried by the “SJ-10” satellite. The orbital height of the satellite is 252 km, the orbital inclination is 42°, and the average daily radiation equivalent dose is 160 µSv. Strains were stored at −20°C after returning.

### 2.2 Growth curves of *E. coli*

*E. coli* carried by the satellite and the ground control group were recovered on the LB solid medium at 37°C overnight. A single colony of each group of *E. coli* was inoculated in 10 mL of LB liquid medium and incubated at 150 rpm at 37°C. Growth curves were obtained by measuring OD_600_ every hour after inoculation for a total of 13 h.

### 2.3 Morphology of *E. coli*

As described in the previous study [7], the effect of spaceflight on the morphology of *E. coli* was studied. Briefly, the preserved strains of *E. coli* were inoculated into the LB solid medium and incubated at 37°C for 5 days to observe the colony wrinkles. To determine the biofilm formation, preserved strains of *E. coli* were recovered with 2 mL LB liquid medium in a 15 mL glass test tube at 37°C and 200 rpm until the exponential phase. OD_600_ of *E. coli* was measured after incubation. The culture medium in the test tube was discarded, and the test tube was washed twice with deionized water. The test tube was placed at 60°C for 1 h and stained with 5 mL 0.1% crystal violet solution for 15 min. The test tube was washed twice with sterile deionized water after discarding the crystal violet solution. Then 5 mL dimethyl sulfoxide was added into the test tube and OD_570_ was measured after 1 h. Relative biofilm production was calculated as 100 × OD_570_/OD_600_. Recovered samples were sent to the China Academy of Chinese Medical Sciences for scanning electron microscope (SEM) analysis with Hitachi S-3400N.

### 2.4 Environmental resistance assay

The environmental resistance assay was done according to the protocol described earlier [7]. Briefly, *E. coli* samples were recovered with the LB liquid medium at 37°C and 200 rpm until the exponential phase. Then samples were added into five mixed LB liquid media (1:100) containing ammonia (pH 9.0), 1% ethanol, 0.0003% hydrogen peroxide, HCl (pH 3.5), or 450 mmol/L NaCl. While the normal LB liquid medium was used as a background. After incubation for 1 h at 37°C and 200 rpm, the number of colonies was counted with the spread plate method. The survival rate was calculated as the number of colonies from the mixed LB medium divided by the number of colonies from the normal LB medium.

### 2.5 Protein extraction for quantitative proteomics

Protein extraction was performed using the protocol described previously with a few modifications [7]. In brief, the preserved strains of *E. coli* were recovered on LB solid medium at 37°C overnight. Single colonies from three technical replicates were inoculated in the LB liquid medium and incubated at 200 rpm at 37°C until the exponential phase (OD_600_ = 1). Samples were harvested by centrifugation at 4,000 rpm for 5 min at 4°C and washed with phosphate buffered saline three times. Then the samples were sonicated on ice for 3 min in lysis buffer containing 8 M urea, 2 mM ethylene diamine tetraacetic acid, 10 mM dithiothreitol (DTT), and 1% protease inhibitor cocktail. After centrifugation at 12,000*g* for 10 min, the supernatant was collected, and the protein concentration was determined by the bicinchoninic acid assay.

### 2.6 TMT labeling

TMT labeling was performed according to the manufacturer’s instructions (Thermo Fisher Scientific, USA) with some modifications [7]. Proteins (100 µg) in each group were reduced with reducing buffer (8 M urea, 10 mM DTT, 100 mM triethylammonium bicarbonate (TEAB), pH 8.0) in Amicon® Ultra-0.5 Centrifugal Filter (10 kDa) at 60°C for 1 h. Then samples were alkylated with iodoacetamide (final concentration of 50 mM) at room temperature in the dark for 40 min. After centrifugation at 12,000 rpm for 20 min, samples were washed three times with 300 mM TEAB and digested with trypsin (Promega, Madison, WI, USA) (enzyme to protein ratio 1:50) in TEAB buffer at 37°C overnight. Peptides from the spaceflight group were labeled with TMT tags 126, 127, and 128. Peptides from control samples were labeled with TMT tags 129, 130, and 131. Aqueous hydroxylamine solution (5% (w/v)) was added to quench the labeling reaction. The six samples were mixed and dried and then resuspended in buffer A (2% acetonitrile and 98% water with ammonia at pH 10) and fractionated to 15 fractions with 1100 HPLC System (Agilent Technologies, USA) coupling with Agilent Zorbax Extend RP column (5 µm, 150 mm × 2.1 mm). Samples were dried and stored at −20℃ until use.

### 2.7 LC-MS/MS analysis

All analyses were performed by a Q-Exactive mass spectrometer (Thermo Fisher Scientific, USA) coupled with a Nanospray Flex source (Thermo Fisher Scientific, USA) as described earlier [7]. Samples were redissolved with 0.1% formic acid and separated by a C18 column (15 cm × 75 µm) on an EASY-nLC^TM^ 1200 system (Thermo Fisher Scientific, USA). The flow rate was 300 nL/min, and the linear gradient was 90 min. Full MS scans were acquired in the mass range of 300–1,600 *m*/*z* with a mass resolution of 70,000, and the automatic gain control (AGC) target value was set at 1e6. The ten most intense peaks in MS were fragmented with higher energy collisional dissociation with positive polarity in the data-dependent mode. MS/MS spectra were obtained with a resolution of 17,500 with an AGC target of 2e5 and a max injection time of 80 ms. Fragmentation was performed with normalized collision energy of 32 and dynamic exclusion of 30 s.

### 2.8 MS data analysis

The MS raw data were analyzed with Maxquant software (version 1.5.2.8) [8] using the Andromeda search engine to search against the UniProt *E. coli* K12 database. The following parameters were applied: isobaric type was selected as 6plex TMT; instrument type was selected as orbitrap with default parameters; the dynamic modifications were oxidation (M) and acetyl (protein N-term); the fixed modification was carbamidomethyl (C); *I* = *L* box was checked; a maximum of two missed cleavages was allowed. Peptides with a minimal length of seven amino acids were considered for protein identification and quantification. A false discovery rate of 0.01 was required for peptides and proteins.

### 2.9 Statistical analysis

Proteins quantified in all samples were considered for further analysis. The log 2 transformed intensity values were normalized with quantile using R (v3.5.3) package limma. Perseus software [9] was used to calculate the differentially expressed proteins with Student’s *t*-test. Permutation-based false discovery rate (FDR) with 250 randomizations was used for multiple hypothesis testing. Proteins with a fold change greater than 1.2 or lower than 5/6 with FDR <5% were significantly changed. The compare groups of growth curves permutation test [10] was used to compare the growth curves of *E. coli* in the spaceflight group and the control group. For other results, the SPSS software package (version 16.0) was used for statistical analysis with an unpaired Student’s *t*-test. The data are presented as means ± SD. All statistic tests were two sided, and statistical significance was set at *P* < 0.05.

## 3 Results

### 3.1 Effect of the space environment on the phenotype of *E. coli*

**Figure 1:**
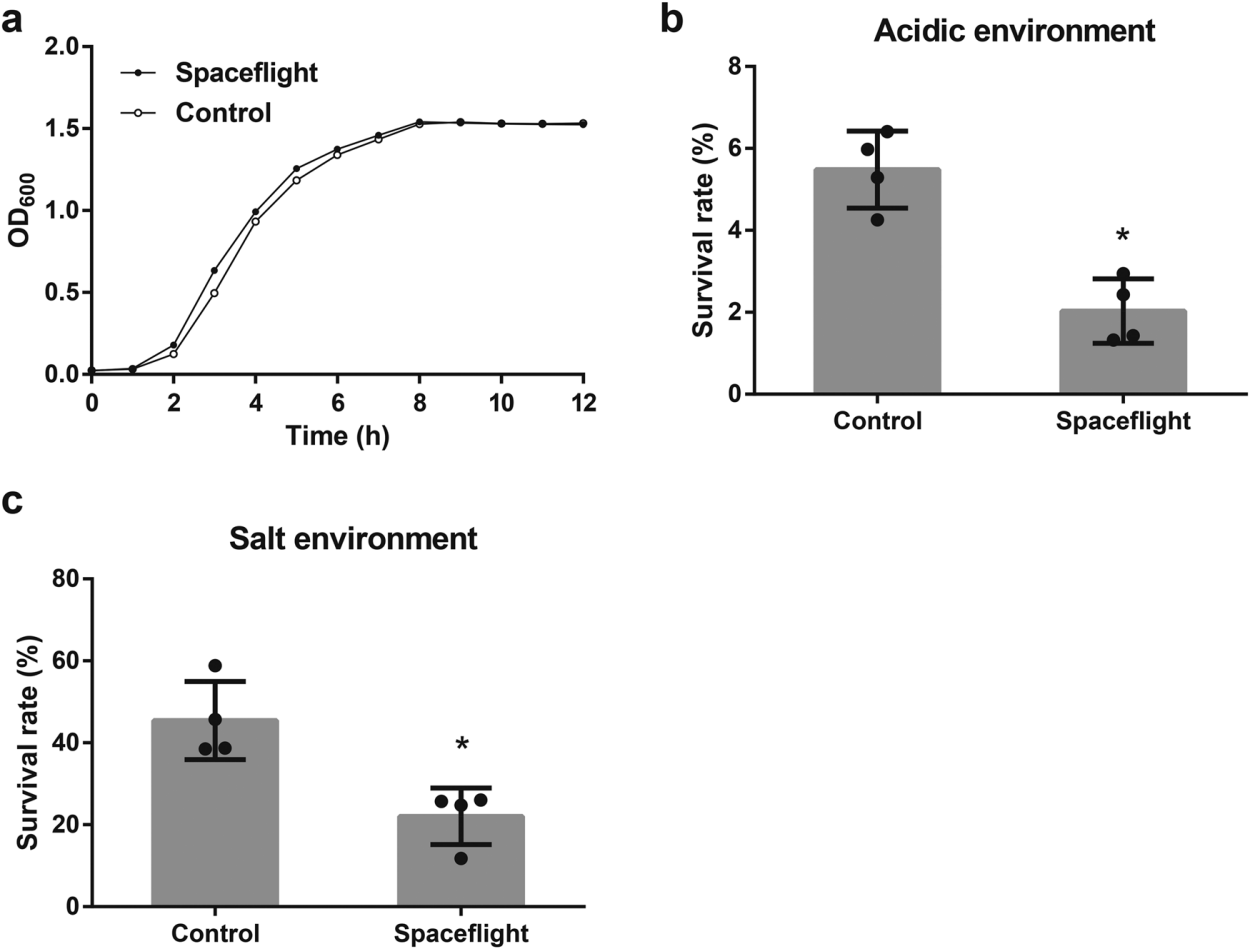
The effect of space environment on the phenotype of *E. coli*. (a) The growth curves of *E. coli* under spaceflight environment compared with control. The survival rate of *E. coli* exposed to (b) HCl (pH 3.5) or (c) 450 mM/L NaCl in the spaceflight group and the control group. *P < 0.05.

**Figure A1:**
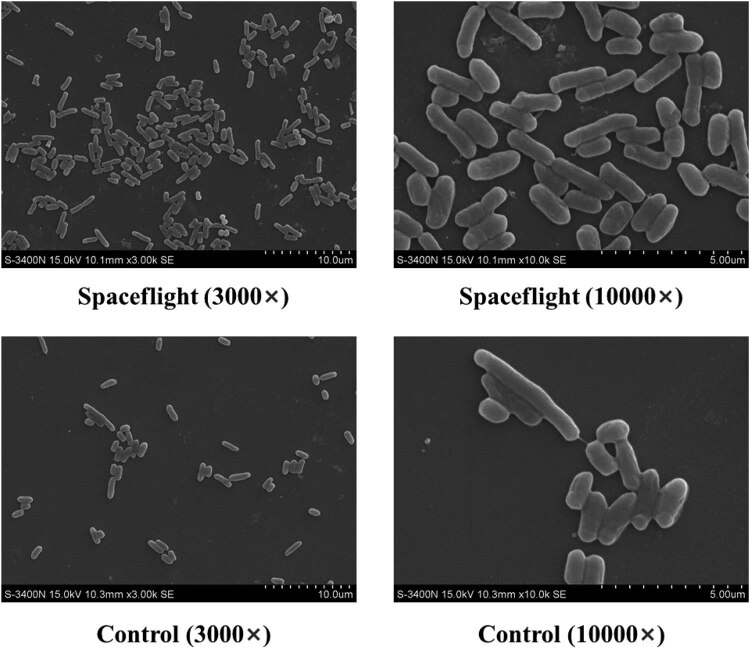
Cell morphology of *E. coli* under control and spaceflight environment.

**Figure A2:**
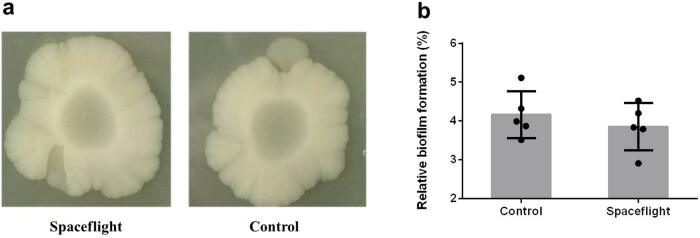
The influence of space environment on *E. coli* biofilm. (a) Colony wrinkles of *E. coli* after being exposed to spaceflight (left) compared with control (right). (b) The relative biofilm formation of *E. coli*.

**Figure A3:**
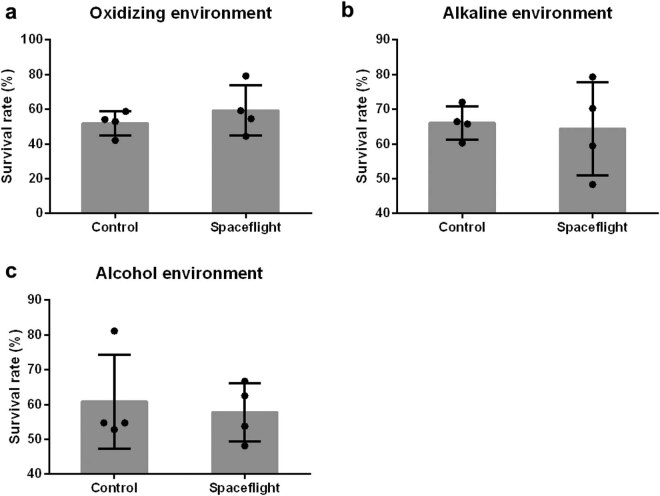
Survival rate of *E. coli* under spaceflight environment compared with control. (a) *E. coli* cultured in LB liquid media with 0.0003% hydrogen peroxide, (b) ammonia (pH 9.0), or (c) 1% ethanol.

After being cultured in space for 12 days, the effect of the space environment on *E. coli* was investigated. We first studied the influence of the space environment on the morphology of *E. coli.* The result of the SEM showed that the morphology of *E. coli* in the spaceflight group has not changed significantly (Figure A1). There is no difference in the formation of wrinkles in the two groups of *E. coli* colonies (Figure A2a). No difference was observed in the relative biofilm formation between the spaceflight group and the control group (Figure A2b). Next, we studied the influence of the space environment on the growth rate of *E. coli*. As shown in Figure 1a, the growth rate of *E. coli* in the spaceflight group was slightly faster than that in the control group. However, there is no significant difference (*P* = 0.1) between the growth curves of the two *E. coli* groups. Finally, we studied the influence of the space environment on the environmental resistance of *E. coli*. The survival rate of *E. coli* in the spaceflight group was lower than that in the control group when cultured in LB medium containing HCl (pH 3.5) or 450 mM/L NaCl (Figure 1b and c). But there is no difference in the survival rate of *E. coli* between the spaceflight group and the control group in resisting hydrogen peroxide, alcohol, and alkaline environments (Figure A3).

### 3.2 TMT-based quantification proteomics for the analysis of *E. coli*

**Figure 2:**
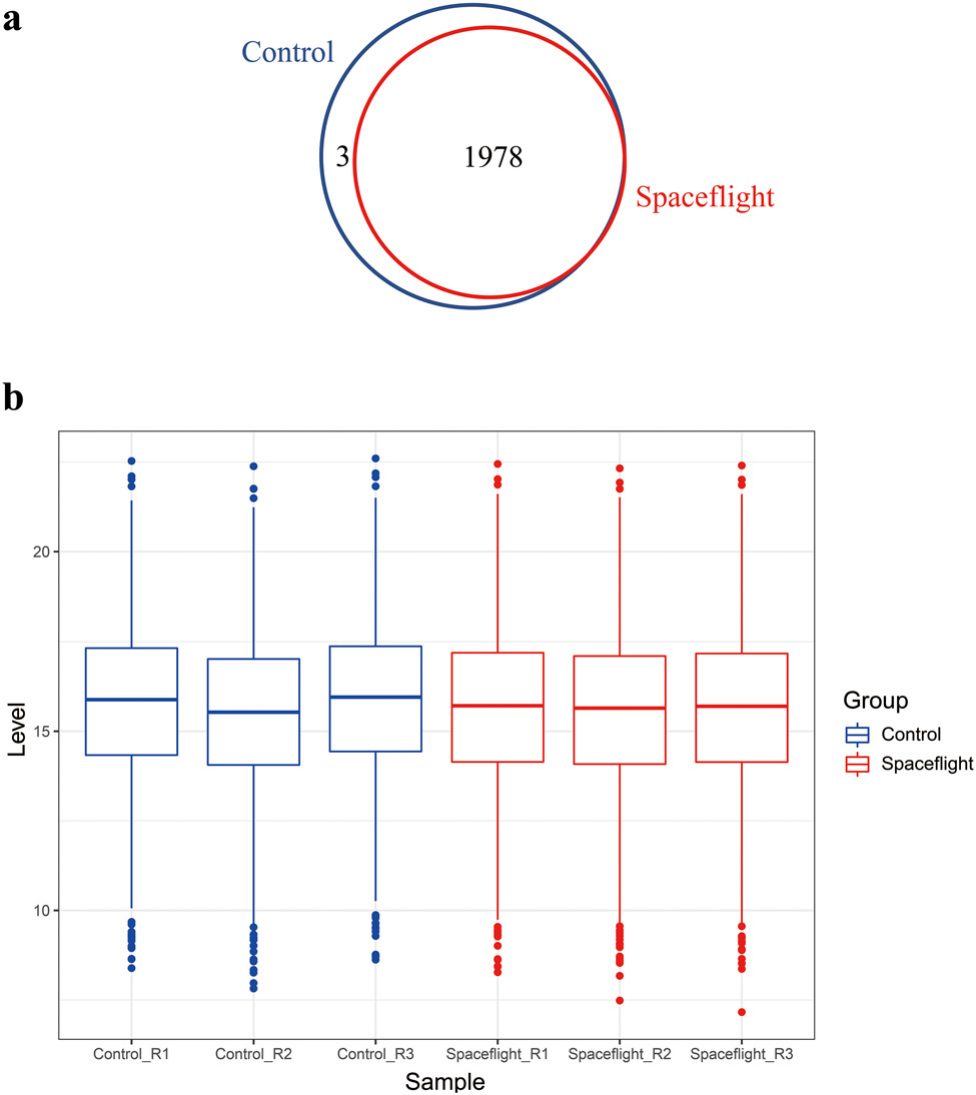
Quantitative analysis of *E. coli* proteome. (a) Venn diagram of protein quantified in controls (blue) and spaceflight group (red). (b) Box plot of protein abundance in each sample.

**Figure 3:**
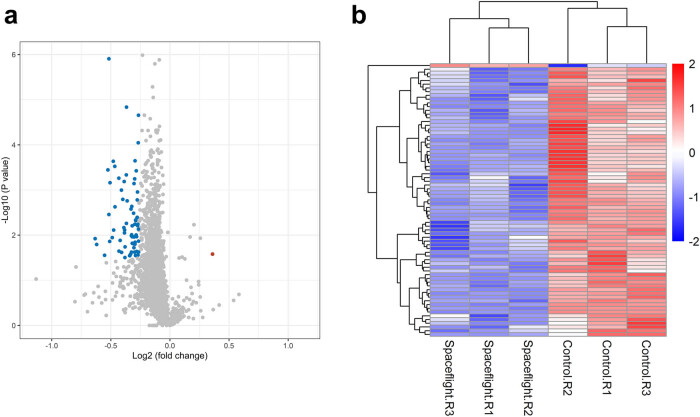
Differentially expressed proteins of *E. coli*. (a) Volcano plot of differentially expressed proteins. The red points represented proteins upregulated in the spaceflight group (FDR < 0.05; fold change ≥1.2). The blue points represented proteins downregulated in the spaceflight group (FDR < 0.05; fold change ≤0.833). (b) Hierarchical clustering of the differentially expressed proteins. The heat map represented the *Z* scores of protein levels across all samples.

Three single colonies of *E. coli* from the spaceflight group and control group were cultured until the exponential phase. Equal amounts of proteins from each sample were digested and labeled with 6-plex TMT. In total, spectral searching identified 1982 protein groups and quantified 1981 protein groups (Figure 2a) with protein and peptide FDR at 0.01. Among them, 1969 protein groups that were quantified in all samples were used for further analysis. The reporter intensity of proteins in each sample was used as the relative quantitative value after log 2 transformed. The distribution of protein abundance in three replicates of the spaceflight sample and three replicates of the control sample showed good consistency of the TMT result (Figure 2b). Student’s *t*-test with permutation-based FDR correction was applied to identify the differentially expressed proteins between spaceflight groups and control groups after quantile normalization. We identified 73 differentially expressed proteins with FDR < 0.05 and ratio > 1.2 (Table S1) or ratio < 0.833 (Table S2). Among them, 72 proteins (98.6%) were downregulated in the spaceflight group compared to the control group (Figure 3a). Hierarchical cluster analysis was performed in Perseus with differentially expressed proteins for all the samples. As shown in Figure 3b, three replicates of the spaceflight sample and three replicates of the control sample were separated clearly as expected. These results demonstrated that the space environment influences the protein expression of *E. coli*. Furthermore, these results support the high reproducibility of our experiments, suggesting that TMT is a reliable quantitative proteomics method.

### 3.3 Effect of the space environment on the biological function of *E. coli*

**Figure 4:**
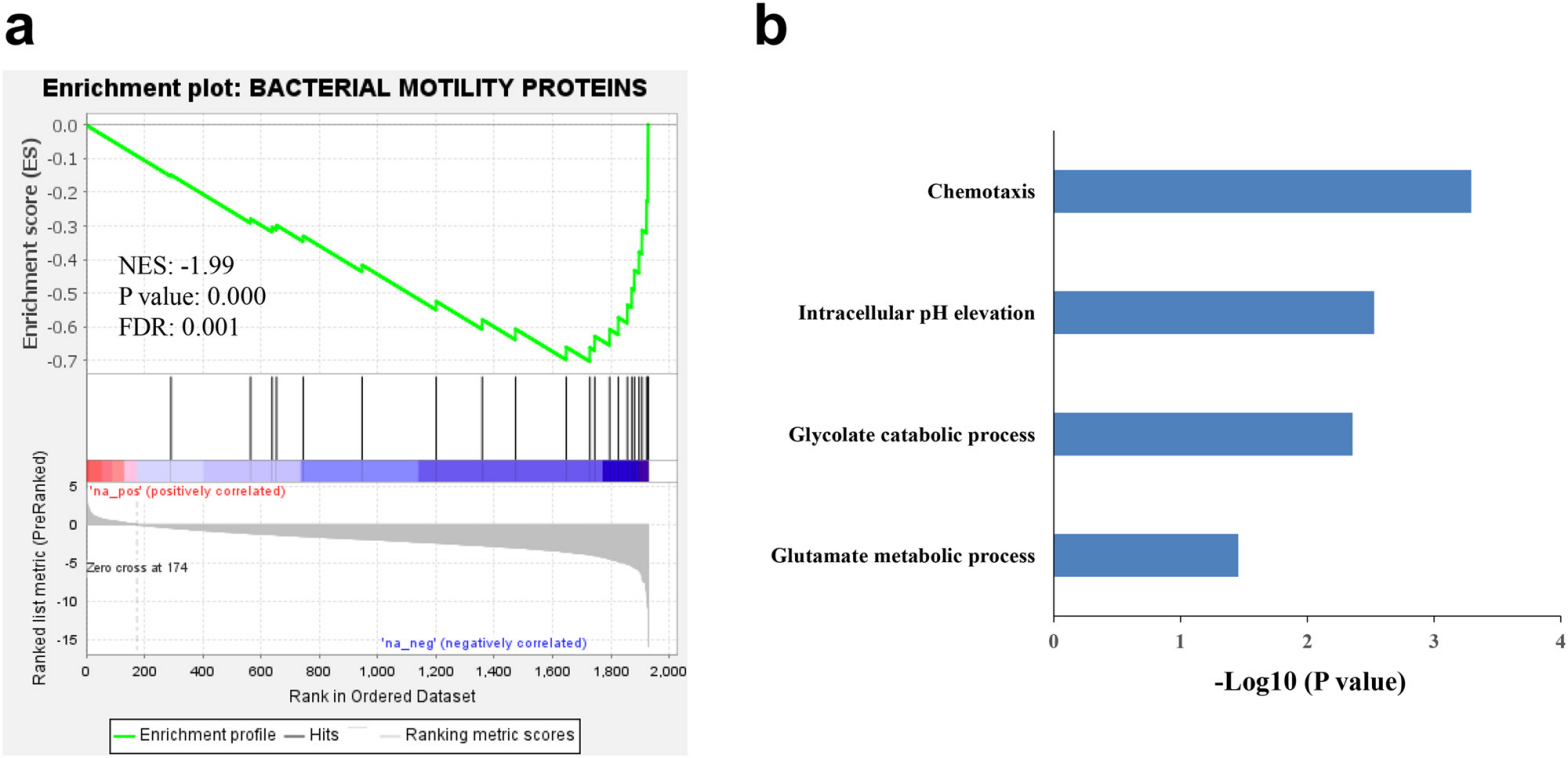
Functional enrichment analysis of *E. coli* proteome. (a) GSEA was performed with the KEGG pathway. (b) Enriched GO biological process with the downregulated proteins in the spaceflight group. The *x*-axis shows the enrichment significance presented with –log 10 (*p*-value).

Functional enrichment analysis was performed to identify the influence of the space environment on the biological function of *E. coli*. On the one hand, gene set enrichment analysis (GSEA) was performed with WebGestalt [11] to enrich the KEGG pathway. Pathways with permutation-based FDR < 0.05 were considered to be enriched in GSEA. As shown in Figure 4a, the bacterial motility proteins pathway was the only enriched KEGG pathway (FDR = 0.001), which was negatively regulated by the space environment (NES = −1.99). On the other hand, overrepresentation analysis (ORA) for the gene ontology (GO) biological process was performed with the differentially expressed proteins by DAVID [12]. Four biological processes including chemotaxis, intracellular pH elevation, glycolate catabolic process, and glutamate metabolic process were significantly enriched with the 72 downregulated proteins in the spaceflight group (Figure 4b). The pathway of intracellular pH elevation helps to maintain a near-neutral intracellular pH when cells are exposed to extremely acidic conditions. The downregulation of this pathway explains the decreased survival rate of *E. coli* in the spaceflight group.

## 4 Discussion

*E. coli* is an opportunistic pathogen, which can cause disease in the small bowels, large bowels, urinary tract, bladder, kidney, and central nervous system under certain conditions [13]. Studies [4] have shown that space flight can cause a decline in the immune function of astronauts. Besides, the space environment may cause changes in microbial pathogenicity. So it is important to understand the phenotype and molecular changes of *E. coli* under space environment. In the present study, we explored the phenotype and profiled the proteomes of *E. coli* after being cultured in space for 12 days. In total, 1982 protein groups were identified, and this is the largest proteomic dataset of *E. coli* under spaceflight conditions to our knowledge.

First, we analyzed the phenotypic of *E. coli*. No difference in growth curves and morphology between the spaceflight group and control group was observed, which was consistent with the previous report [5,14]. At the same time, there was no difference in the survival rate of *E. coli* against the hydrogen peroxide, alcohol, and alkaline environments between the two groups. The associated proteins were also not significantly different between the two groups. For example, OxyR protein, which is responsible for hydrogen peroxide resistance in *E. coli* [15], was not differentially expressed between the two groups. However, we found that the survival rate of *E. coli* in the spaceflight group was lower than that in the control group under acidic condition and hyperosmotic stress. To explore the mechanism of this phenotype, we analyzed the proteome result and found that five proteins involved in acid resistance and two proteins involved in osmotic pressure adjustment were downregulated in the spaceflight group. Among the five acid-resistance proteins, gadA and gadB are two types of glutamate decarboxylase that convert glutamate into gamma-aminobutyrate (GABA) [16]. gadC is involved in the transport of glutamate and GABA under acidic conditions [17]. The gad system, which protects cells from acidic conditions, was downregulated in the spaceflight group. In addition, the levels of hdeB and ydeP, which are important for the survival of *E. coli* in the acidic environment, were also decreased in the spaceflight group. Both proteomics results and phenotypic analysis showed that the acid resistance of *E. coli* in the spaceflight group was decreased. Yim et al. [18] found that microgravity decreased the acid resistance of *E. coli* Nissle 1917, which was consistent with our results. However, Zea et al. [19] observed an upregulation of acid resistance-related genes in *E. coli* ATCC 4157 after being cultured in a medium containing Gentamicin Sulfate in the International Space Station. The difference in these results may be caused by different experimental conditions such as strain, medium composition, spaceflight time, and environment. Consistent with the decline in the survival rate of *E. coli* in the spaceflight group under high salt conditions, the level of osmY that involved in the adaptation to hyperosmotic stress [20] was downregulated. Interestingly, low conductance mechanosensitive channel YnaI (ynaI) that protects cells against hypoosmotic stress [21] was also decreased in the spaceflight group. These results indicate that *E. coli* in the spaceflight group has a reduced ability to survive under extremely osmotic stress.

Next, we analyzed the biological function changes of *E. coli* under space environment. GO biological process enrichment analysis showed that proteins in chemotaxis, intracellular pH elevation, glycolate catabolic process, and glutamate metabolic process were significantly downregulated in the spaceflight group. Proteins enriched in the glutamate metabolic process, and intracellular pH elevation belongs to the gad system, which is related to acid resistance [16], indicating that the biological function changes of *E. coli* after exposure to the space environment are consistent with the phenotype result. Chemotaxis was the most significant biological process of enrichment analysis. Six proteins including fliG, fliM, cheA, cheB, tar, and tsr related to chemotaxis were downregulated in the spaceflight group. In addition, the bacterial motility protein pathway was enriched in GSEA, which is also related to chemotaxis. Our results indicate that the chemotaxis of *E. coli* in the spaceflight group was suppressed. However, different conclusions have been found in other similar studies. Tucker et al. [22] cultured *E. coli* MG1655 in minimal (3-(N-morpholino)propanesulfonic acid (MOPS) + glucose) medium and LB medium under a low-shear modeled microgravity (LSMMG) environment produced by a high-aspect rotating vessel. They found that genes involved in the *E. coli* acid tolerance response (*gadE*, *hdeB*), cell motility (*flg* and *fli* genes), and chemotaxis (*cheZ, tar*) were upregulated in LSMMG compared to the control under minimal MOPS medium. But no changes of these genes were observed in LSMMG compared to normal gravity controls under the LB medium. Only 14 downregulated genes were identified in LSMMG compared to the control under the LB medium. Those results and many other studies [18,19,23,24,25] demonstrated that the different space vehicles, bacterial strains, culture medium, and other factors can affect the response of *E. coli* to microgravity or spaceflight.

Tryptophan-specific transport protein (mtr) was the only upregulated protein in *E. coli* of the spaceflight group. This protein is involved in transporting tryptophan into the cell [26]. Our results showed that tryptophan is extremely important for *E. coli* in responding to the space environment. Interestingly, we found that other proteins with an upregulation trend (FDR < 0.05, 1 < ratio < 1.2) in the spaceflight group were all related to pyrimidine biosynthesis. In this pathway, carA (ratio = 1.15) and carB (ratio = 1.07) synthesize carbamoylphosphate from glutamine [27]. Then aspartate carbamoyltransferase catalyzes the conversion of carbamoylphosphate and aspartate to carbamoylaspartate [27]. PyrB (ratio = 1.13) is the catalytic subunit of aspartate carbamoyltransferase, and pyrI (ratio = 1.19) is the regulatory subunit of aspartate carbamoyltransferase. However, other enzymes of pyrimidine biosynthesis did not show an upregulation trend in the spaceflight group. The role of cumulative carbamoylaspartate in the spaceflight group of *E. coli* needs further study.

## 5 Conclusions

Our research explored the changes in the phenotype and proteome of *E. coli* after exposure to the space environment. The results of the phenotypic experiment showed that the survival rate of *E. coli* in the spaceflight group decreased in acidic and high-salt environments. Proteomics analysis revealed 73 differentially expressed proteins in the spaceflight group compared to the control group. Functional enrichment analysis showed that proteins in chemotaxis, intracellular pH elevation, glycolate catabolic process, and glutamate metabolic process were significantly downregulated in the spaceflight group. These results indicate that proteomics data can explain phenotypic changes, and our research will provide a theoretical basis for microbial safety in the space environment.

## Supplementary Material

Supplementary Table
