# Supplementary material for: Effect of spaceflight on the phenotype and proteome of Escherichia coli
Source: Open Life Sci. 2023 Feb 28;18(1):20220576. doi: 10.1515/biol-2022-0576 (PMC9975951; doi:10.1515/biol-2022-0576)
Supplement: Supplementary Table [file biol-2022-0576-sm.pdf]

Supplementary material

Table S1: Upregulated proteins in *Escherichia coli* after spaceflight

Accession	Gene names	Protein names	*P*-value	FDR	Fold change (spaceflight/control)
P0AAD2	mtr	Tryptophan-specific transport protein	2.63×10^{-2}	4.32×10^{-2}	1.284

Table S2: Downregulated proteins in *Escherichia Coli* after spaceflight

Accession	Gene names	Protein names	*P*-value	FDR	Fold change (spaceflight/control)
P64588	yqjI	Transcriptional regulator YqjI	2.37×10^{-2}	3.96×10^{-2}	0.832
P06961	cca	Multifunctional CCA protein	1.36×10^{-2}	2.41×10^{-2}	0.832
P33029	yeiQ	Uncharacterized oxidoreductase YeiQ	2.21×10^{-5}	0.00×10	0.832
P23908	argE	Acetylornithine deacetylase	7.36×10^{-3}	1.68×10^{-2}	0.831
P0AFH8	osmY	Osmotically-inducible protein Y	8.99×10^{-5}	0.00×10	0.83
P0ADS2	zapA	Cell division protein ZapA	2.77×10^{-2}	4.51×10^{-2}	0.829
P42604	uxaA	Altronate dehydratase	5.75×10^{-3}	1.42×10^{-2}	0.829
P65807	ygeY	Uncharacterized protein YgeY	2.75×10^{-2}	4.42×10^{-2}	0.828
P0A9H7	cfa	Cyclopropane-fatty-acyl-phospholipid synthase	6.14×10^{-3}	1.51×10^{-2}	0.827
P0ABZ1	fliG	Flagellar motor switch protein FliG	1.11×10^{-3}	6.19×10^{-3}	0.827
P64476	ydiH	Uncharacterized protein YdiH	1.66×10^{-3}	8.00×10^{-3}	0.826
P37672	dlgD	2,3-Diketo-L-gulonate reductase	4.04×10^{-3}	1.09×10^{-2}	0.825
P0ADK8	yibL	Uncharacterized protein YibL	4.14×10^{-3}	1.08×10^{-2}	0.824
P42593	fadH	2,4-Dienoyl-CoA reductase	2.89×10^{-2}	4.58×10^{-2}	0.823
P0ABN9	dcuB	Anaerobic C4-dicarboxylate transporter DcuB	2.30×10^{-2}	3.89×10^{-2}	0.823
P76104	rlhA	23S rRNA 5-hydroxycytidine C2501 synthase	1.08×10^{-2}	2.02×10^{-2}	0.822
P05804	uidA	Beta-glucuronidase	3.76×10^{-4}	1.80×10^{-3}	0.82
P42616	yqjC	Protein YqjC	9.93×10^{-3}	2.03×10^{-2}	0.82
P0AF24	nagD	Ribonucleotide monophosphatase NagD	4.78×10^{-3}	1.04×10^{-2}	0.819
P32162	yiiS	UPF0381 protein YiiS	4.66×10^{-3}	1.05×10^{-2}	0.817
P0ACI6	asnC	Regulatory protein AsnC	2.82×10^{-2}	4.48×10^{-2}	0.817
P0A7J0	ribB	3,4-Dihydroxy-2-butanone 4-phosphate synthase	2.25×10^{-4}	0.00×10	0.815
P0AA78	exuT	Hexuronate transporter	1.44×10^{-2}	2.47×10^{-2}	0.815
P0A9A2	ftnB	Bacterial non-heme ferritin-like protein	1.58×10^{-2}	2.59×10^{-2}	0.815
P0A6X1	hemA	Glutamyl-tRNA reductase	5.76×10^{-3}	1.41×10^{-2}	0.814
P0ACD8	hyaB	Hydrogenase-1 large chain	5.67×10^{-4}	5.43×10^{-3}	0.813
P07330	cheB	Protein-glutamate methylesterase/protein-glutamine glutaminase	2.37×10^{-2}	3.95×10^{-2}	0.813
P67603	yqfB	N	1.10×10^{-2}	2.01×10^{-2}	0.81
P0A9H3	cadA	Inducible lysine decarboxylase	7.20×10^{-4}	4.85×10^{-3}	0.81

(*Continued*)

Table S2: *Continued*

Accession	Gene names	Protein names	*P*-value	FDR	Fold change (spaceflight/control)
P0AD53	ygaC	Uncharacterized protein YgaC	1.90×10^{-2}	3.20×10^{-2}	0.81
P75780	fiu	Catecholate siderophore receptor Fiu	1.56×10^{-2}	2.62×10^{-2}	0.808
P75825	hcp	Hydroxylamine reductase	3.27×10^{-3}	9.85×10^{-3}	0.808
P46118	hexR	HTH-type transcriptional regulator HexR	1.52×10^{-2}	2.65×10^{-2}	0.802
P0A9E5	fnr	Fumarate and nitrate reduction regulatory protein	6.21×10^{-3}	1.50×10^{-2}	0.801
P77615	ycjW	Uncharacterized HTH-type transcriptional regulator YcjW	1.09×10^{-2}	2.00×10^{-2}	0.801
P21169	speC	Constitutive ornithine decarboxylase	2.31×10^{-3}	8.56×10^{-3}	0.799
P62672	apaG	Protein ApaG	9.81×10^{-3}	2.05×10^{-2}	0.799
P0CK95	yghJ	Putative lipoprotein AcfD homolog	2.38×10^{-2}	3.93×10^{-2}	0.798
P77611	rsxC	Ion-translocating oxidoreductase complex subunit C	1.02×10^{-2}	2.00×10^{-2}	0.798
P37636	mdtE	Multidrug resistance protein MdtE	2.64×10^{-3}	8.54×10^{-3}	0.796
P0AAK7	nrfC	Protein NrfC	2.63×10^{-2}	4.33×10^{-2}	0.794
P0ADB7	ecnB	Entericidin B	2.85×10^{-2}	4.51×10^{-2}	0.79
P0AC16	folB	Dihydroneopterin aldolase	2.85×10^{-2}	4.52×10^{-2}	0.789
P77561	ydeP	Protein YdeP	1.74×10^{-3}	7.74×10^{-3}	0.778
P07017	tar	Methyl-accepting chemotaxis protein II	1.46×10^{-5}	0.00×10	0.775
P43341	lpxH	UDP-2,3-diacylglucosamine hydrolase	5.49×10^{-3}	1.38×10^{-2}	0.774
P0ADI4	entB	Enterobactin synthase component B	4.62×10^{-4}	3.86×10^{-3}	0.774
P0AEP9	glcD	Glycolate oxidase subunit GlcD	1.82×10^{-2}	2.97×10^{-2}	0.769
P45807	ybaM	Uncharacterized protein YbaM	3.14×10^{-2}	4.88×10^{-2}	0.768
P06974	fliM	Flagellar motor switch protein FliM	7.69×10^{-3}	1.77×10^{-2}	0.767
P0AAA9	zraP	Zinc resistance-associated protein	8.92×10^{-3}	1.99×10^{-2}	0.766
P08555	dsdX	D-serine transporter DsdX	6.49×10^{-4}	5.06×10^{-3}	0.765
P17315	cirA	Colicin I receptor	6.81×10^{-3}	1.56×10^{-2}	0.763
P63235	gadC	Glutamate/gamma-aminobutyrate antiporter	1.61×10^{-3}	8.28×10^{-3}	0.758
P77454	glsA1	Glutaminase 1	2.41×10^{-2}	4.09×10^{-2}	0.753
P76227	ynjH	Uncharacterized protein YnjH	1.01×10^{-3}	6.59×10^{-3}	0.752
P60869	ybjL	Putative transport protein YbjL	1.31×10^{-2}	2.43×10^{-2}	0.747

(*Continued*)

Table S2: *Continued*

Accession	Gene names	Protein names	*P*-value	FDR	Fold change (spaceflight/control)
P52644	hslJ	Heat shock protein HslJ	2.26×10^{-2}	3.86×10^{-2}	0.742
P02942	tsr	Methyl-accepting chemotaxis protein I	5.47×10^{-4}	5.60×10^{-3}	0.741
P77161	glxR	2-Hydroxy-3-oxopropionate reductase	2.36×10^{-3}	8.37×10^{-3}	0.724
P69910	gadB	Glutamate decarboxylase beta	3.00×10^{-4}	0.00×10	0.724
P08202	araA	L-Arabinose isomerase	7.73×10^{-3}	1.75×10^{-2}	0.719
P07363	cheA	Chemotaxis protein CheA	2.31×10^{-4}	0.00×10	0.717
P0AEB5	ynaI	Low conductance mechanosensitive channel YnaI	1.13×10^{-2}	2.15×10^{-2}	0.713
P0AEP7	gcl	Glyoxylate carboligase	6.89×10^{-4}	4.99×10^{-3}	0.704
P00926	dsdA	D-Serine dehydratase	1.38×10^{-2}	2.39×10^{-2}	0.702
P77671	allB	Allantoinase	1.24×10^{-6}	0.00×10	0.699
P0AET2	hdeB	Acid stress chaperone HdeB	3.49×10^{-3}	1.04×10^{-2}	0.699
P0AE58	caiF	Transcriptional activatory protein CaiF	3.59×10^{-4}	1.88×10^{-3}	0.695
P0A843	tatE	Sec-independent protein translocase protein TatE	2.80×10^{-2}	4.49×10^{-2}	0.682
P64493	yoaF	Uncharacterized protein YoaF	1.61×10^{-2}	2.58×10^{-2}	0.651
P69908	gadA	Glutamate decarboxylase alpha	1.19×10^{-2}	2.29×10^{-2}	0.645
